# Une trichotillomanie sévère associée à des obsessions idéatives

**Published:** 2012-12-18

**Authors:** Souad Rharrabti, Rachid Aalouane

**Affiliations:** 1 Service de psychiatrie, CHU Hassan II, Fès, Maroc

**Keywords:** Trichotillomanie, obsessions idéatives, syndrome dépressif, psychothérapie, trichotillomania, obsessive ideation, depressive syndrome, psychotherapy

## Images in médicine

La trichotillomanie est une pathologie quasiment féminine touchant selon les études 0,5 à 2% de la population. Elle consiste en une conduite compulsives d'arrachage des cheveux, des sourcils ou tout autres poils survenant plus fréquemment en fin de journée, ce qui entraîne des plaques d'alopécie parfois très étendues. Elle est classée comme un trouble de contrôle des impulsions selon la classification de DSMIV (Diagnostic and Statistical Manual of Mental Disorders). Nous rapportons le cas d'une patiente âgée de 27ans, célibataire, sans antécédents psychiatriques notables, qui présente depuis cinq ans une symptomatologie faite d'un isolement avec des crises de pleurs accompagné d'une trichotillomanie. La patiente a arraché ses cheveux, ses cils et sourcils de façon incontrôlée avec recrudescence le soir, ceci est associé à une insomnie, une anorexie, un désintérêt et une perte de plaisir. L'examen psychiatrique a trouvé un syndrome dépressif franc, des idées obsessionnelles de type idéatives. L'examen physique a trouvé des plaque d'alopécie très étendues avec cheveux très courts, les cils sont rares et les sourcilles sont quasiment arrachés, quelques lésions d'excoriations sur la face postérieure des mains. La patiente a été mise sous valproate de sodium à la dose de 400mg associé à la sertraline à la dose de 100mg. Une prise en charge psychothérapique cognitivo-comportementale était débuté. On a noté une disparition des symptômes dépressifs et une légère atténuation du comportement de trichotillomanie.

**Figure 1:**
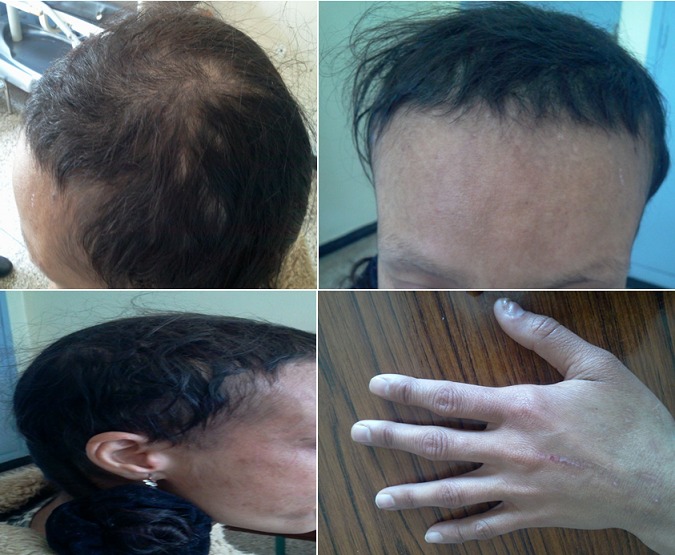
Images montrant des plaques d'alopécie, cheveux courts, sourcilles arrachés, cicatrices de lésions d'excoriation sur la face dorsale des mains

